# Spatially fractionated radiation therapy for treating recurrent glioblastoma: a dosimetric feasibility study

**DOI:** 10.3389/fonc.2025.1691132

**Published:** 2026-01-05

**Authors:** Yuwei Zhou, Sean Tanny, Michael T. Milano, Brian Marples, Fiona Li, Hyunuk Jung, Matthew Webster, Alexander R. Podgorsak, Jihyung Yoon, Wesley Rivais, Michael J. Hazoglou, Dandan Zheng

**Affiliations:** Department of Radiation Oncology, University of Rochester Medical Center, Rochester, NY, United States

**Keywords:** EQD2, lattice, recurrent GBM, SCART, SFRT

## Abstract

**Objective:**

Spatially fractionated radiation therapy (SFRT) shows promise for treating bulky, advanced, or recurrent tumors. To evaluate the feasibility of SFRT for patients with recurrent glioblastoma (GBM), we conducted a planning study involving 14 patients, analyzing vertex target volume (VTV) contours and cumulative doses to both targets and organs at risk (OARs).

**Method:**

The patients were divided into two groups based on gross tumor volume (GTV): 10 patients with GTV > 15 cc; 4 patients with GTV ≤ 15 cc. SFRT was planned as an upfront boost, using LATTICE radiotherapy (LRT) and stereotactic central ablative radiation therapy (SCART) respectively. With a LRT technique, vertex diameters ranged from 0.8–1.5 cm, with center-to-center spacing of 2–4 cm.

**Result:**

GTV geometry—not size—determined mean vertex diameter (MVD: 0.99 ± 0.12 cm), spacing (2.93 ± 0.34 cm), and the VTV-to-GTV ratio (VGR: 6.6 ± 1.7%). With a SCART technique, the mean VGR was 25.8 ± 10.0%. Compared with the original sum plan, the cumulative EQD2_2_ dose in the SFRT sum plan to critical OARs was well-controlled, such as the brainstem with a difference of 0.36 ± 1.00%. However, V120Gy to the brain in the SFRT sum plan increased by 4.51 ± 3.97 cc, for the 12 patients with an original V120Gy < 2 cc. Increased V120Gy to the brain might elevate the risk of radiation-induced necrosis.

**Conclusion:**

In summary, our planning study demonstrates that dosimetrically acceptable SFRT plans can be achieved for recurrent GBM. The main clinical consideration is balancing the potential benefit of SFRT against the risk of radiation-induced necrosis.

## 1 Introduction

Spatially fractionated radiation therapy (SFRT) was originally developed to treat bulky tumors while reducing skin toxicity (1). With the advent of advanced radiation techniques, there has been renewed interest in the use of SFRT (2). Its hallmark feature is the delivery of radiation in a deliberately heterogeneous dose pattern. SFRT can be implemented through several subtypes—such as GRID therapy, LATTICE radiotherapy (LRT), and stereotactic central ablative radiation therapy (SCART) (3–7). Both GRID therapy and LRT have demonstrated clinical success in the management of metastatic and recurrent disease, as well as in selected non-metastatic primary tumors, including cervical cancer, soft tissue sarcomas, and non–small cell lung cancer (NSCLC) (2, 8–11). Demonstrating tolerable toxicity and high tumor response rates in the management of extracranial bulky tumors, SFRT provides a strong rationale for its translation to cranial applications (8–10).

Recent experience with single isocenter, multi-metastasis stereotactic radiotherapy (12), along with successful SFRT outcomes, suggests that highly heterogeneous dose distributions may offer greater efficacy and tolerability for large cranial targets compared with conventional radiotherapy. In SFRT planning, the ablative dose is directed to a portion of the planning target volume (PTV) while sparing critical organs at risk (OARs), thereby reducing radiation toxicity to surrounding healthy tissue. The high “peak” dose may enhance tumor control through mechanisms such as the bystander effect, vascular damage, and immune activation (13–16), whereas the low “valley” dose helps preserve vascular structures around high-dose regions, maintaining perfusion and enabling the transport of tumoricidal cells and cytokines (6). Recent studies have demonstrated that SFRT can further enhance immune responses – including cohort, bystander, and abscopal effects – compared with conventional radiotherapy (17, 18). For bulky tumors, this approach may provide advantages over conventionally prescribed radiation therapy.

Given its success in extracranial applications, extending SFRT to cranial lesions is a logical step. However, due to the potential risks that can be associated with brain irradiation, preclinical and early clinical feasibility studies are essential before broader clinical adoption. Glioblastoma (GBM) is one of the most aggressive malignancies. Higher radiation dose could potentially offer better control, though it is limited by risks of treatment-related toxicity (i.e. dose to the OARs). Thus, GBM – particularly GBM recurrent after prior radiotherapy – presents a compelling target for an investigation of SFRT, as these patients often experience severe toxicity with tumoricidal doses delivered via conventional reirradiation (19). The superior ability of SFRT to spare normal tissue while delivering high doses to the tumor makes it a feasible and generally well-tolerated approach for re-irradiation, particularly in the palliative setting (20). An upfront SFRT boost may allow delivery of a more effective dose while maintaining tolerability. This study evaluates the dosimetric feasibility of SFRT in patients with recurrent GBM, aiming to support its potential role as a treatment option when conventional RT outcomes are suboptimal.

## 2 Materials and methods

### 2.1 Patient selection

This IRB-approved study utilized the reporting tool of Varian ARIA^®^ (Varian Medical Systems, Palo Alto, CA, USA) to identify patients treated at our institution for GBM over the past 10 years. From this dataset, we screened for patients with recurrent GBM who had undergone at least two courses of brain radiotherapy. Each case was reviewed, and patients whose gross tumor volume (GTV) in the most recent treatment course was less than 3 cc were excluded since SFRT is more advantageous for larger tumors.

A total of 14 patients with recurrent GBM met the inclusion criteria. Based on the GTV in their latest treatment course, patients were further divided into two groups:

Group 1: 10 patients with GTV > 15 cc.

Group 2: 4 patients with GTV ≤ 15 cc.

### 2.2 SFRT target structure contour

A retrospective planning study was conducted in Varian Eclipse^®^ (Varian Medical Systems, Palo Alto, CA, USA) to evaluate the feasibility of SFRT. The SFRT plan was created based on the recurrent treatment (2nd course). For Group 1 patients, SFRT vertices were initially generated within the high-risk planning target volume (PTV) using a published script with parameters optimized for this study (4 mm vertex radius and 20–25 mm spacing), and then manually adjusted to target up to 10% of the GTV (21). Vertex placement was refined to remain within the GTV whenever possible, maintaining an initial center-to-center spacing of ~2.5 cm (adjustable to 2–4 cm based on dosimetric evaluation). Vertices with an initial 8 mm diameter were expandable up to 15 mm to cover a larger portion of the GTV without exceeding GTV boundaries. The total volume of all vertices was defined as the vertex target volume (VTV). Further VTV modifications could be performed based on dosimetric evaluation, as described in Section 2.3.

For Group 2 patients, the GTVs were too small to allow multiple vertices with adequate spacing; thus, only a single central vertex could be placed. The treatment strategy of SCART can be viewed as a form of simultaneous integrated boost: the SFRT dose is prescribed to the whole tumor, with the peak dose concentrated in the tumor core and the valley dose delivered to the tumor edge. In this SCART planning group, instead of a spherical vertex, a reduced partial volume derived from the GTV or PTV was used as the VTV. If the margin between the PTV and GTV exceeded 3 mm, the VTV was generated by reducing the GTV margin by 2 mm. If the margin was ≤ 3 mm, the VTV was generated from the PTV by reducing the margin by 5 mm. This approach ensured a minimum separation of > 5 mm between the PTV and VTV, thereby minimizing high-dose spillage to normal brain tissue.

### 2.3 SFRT planning

SFRT plans were prescribed to deliver in a single fraction targeting both the VTV and PTV. A peak dose of 15 Gy was prescribed to fully cover the VTV, with the valley dose was limited to < 5 Gy. The PTV prescription matched that of the 2nd course but was capped at 4 Gy per fraction. If the original 2nd course PTV dose per fraction exceeded 5 Gy, it was scaled down to 4 Gy.

Plans were created in Eclipse^®^ using 4–7 non-coplanar semi-arcs (6 MV flattening-filter-free beams) on a Varian Edge^®^ equipped with an HD-MLC (Varian Medical Systems, Palo Alto, CA, USA). To evaluate valley dose and guide optimization, a ring structure was created from the VTV with an outer margin of 10 mm and an inner margin of –5 mm. A key planning goal was to minimize overlapping 5 Gy coverage between vertices, with the objective of keeping V5Gy of the ring structure below 10–12%.

For Group 1 patients, if this goal could not be achieved through beam optimization alone, the VTV was iteratively modified by resizing and repositioning vertices to increase spacing and improve dose fall-off. Vertices were preferentially moved within the GTV, avoiding critical OARs. If OAR dose constraints were unmet, the VTV was cropped to fit within the GTV plus a 2 mm margin (GTV_2mm) to reduce normal brain dose, maintaining at least 1 cm clearance from critical OARs such as the brainstem. After 2–3 iterations of adjustment, final SFRT plans achieved ≥ 95% VTV coverage at 15 Gy, maintained a valley dose < 5 Gy, and met all dose constraints of the 2nd conventional RT course. With a margin greater than 5 mm between the PTV and VTV, the valley dose objective was achievable for Group 2 without additional modifications.

### 2.4 Data analysis

SFRT plan parameters—including vertex diameter, spacing, diameter-to-spacing ratio, VTV to GTV ratio (VGR), and peak to valley dose ratio (PVDR)—were analyzed statistically. For Group 1 patients, PVDR was defined as the ratio of D5% to D95% within the GTV. For Group 2 patients, as the D95% of the GTV in all plans exceeded 5 Gy, PVDR was evaluated using the PTV instead.

To assess dose coverage for the PTV and GTV, the first fraction of the 2nd course was replaced with the SFRT plan to create an SFRT course (upfront SFRT boost + conventional RT with n-1 fractions). The SFRT sum plan was defined as the composite equivalent dose in 2 Gy fractions (EQD2) of the 1st course and the SFRT course. The original sum plan was the composite EQD2 of the 1st course and the original 2nd course. In both sum plans, the 2nd course planning CT and SFRT plan structure set were used.

For EQD2 calculations, the GTV_2mm structure—generated by expanding the GTV by a 2 mm margin—was assigned an α/β ratio of 8 (22). For the 14 retreatment cases, the CTV+PTV margin ranged from 2 to 15 mm. To better assess the dose to potential subclinical disease and to account for minimal setup uncertainty, using GTV_2mm is more appropriate for calculating the BED. For the Brain–GTV_2mm structure (brain minus GTV_2mm), brainstem, and optic chiasm, an α/β ratio of 2 was used (23).

Cumulative EQD2 values for both sum plans were analyzed for target volumes and OARs, including normal brain (brain minus GTV), brainstem, and optic chiasm. Comparisons between the original sum plan (1st course + 2nd course) and the SFRT sum plan were performed. In addition, dose–volume histogram (DVH) gamma analysis (DVH γ) with 1%/1% criteria was applied for critical OARs, where 1%/1% refers to 1% of the SFRT sum plan maximum dose and 1% of the respective structure’s volume (24).

## 3 Results

Most patients received their 1st course of treatment with the standard prescription of 60 Gy in 30 fractions. In contrast, the 2nd course varied, ranging from 25 Gy in 5 fractions to 59.4 Gy in 33 fractions. Details of the original prescriptions and recurrence pattern for each patient are provided in **Supplementary Table S1** (25). The 2nd course GTV sizes ranged from 3.7 to 117.8 cc, with coverage exceeding 95% (**Supplementary Tables S2, S3**).

### 3.1 SFRT plan evaluation

The optimized vertex radius and spacing for script-based generation were 4 mm and 21–25 mm, respectively. While the script provided a reasonable initial vertex distribution, further manual modification was required. **Supplementary Table S2** summarizes the optimized vertex and SFRT plan parameters for each patient.

**Figure 1:**
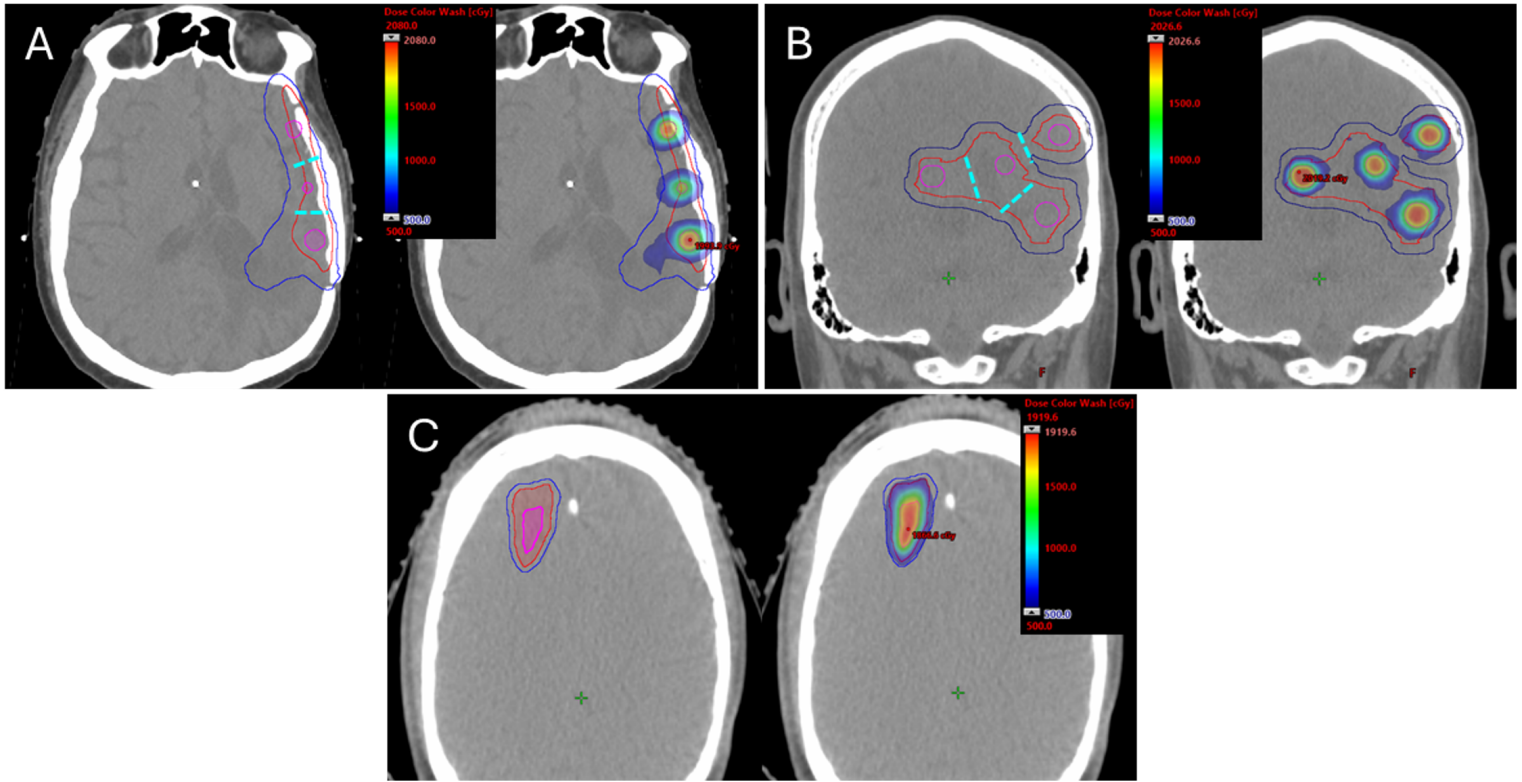
VTV (magenta), GTV (red), PTV (blue), and dose maps for P1’s **(A)**, P5’s **(B)** and P12’s **(C)** SFRT plans. Cyan dashed lines indicated the separation between vertices. The dose color wash range was 500–2000 cGy. Compared with P5, the GTV segments of P1 were narrower, resulting in smaller vertices and VTV. Due to the shorter vertex spacing in P1, the dose distribution around the vertices spread more laterally. For the same reason, a few vertices in P1 were not entirely contained within the GTV, resulting in a 36.4% increase in normal brain dose, compared with 4.9% for P5, where the VTV was fully within the GTV. For P12, the VTV was derived from the PTV by reducing the margin by 5 mm to ensure a dose fall-off to 5 Gy at the PTV edge.

For Group 1 Patients, the mean vertex diameter (MVD) was 9.9 ± 1.2 mm, with a mean vertex diameter-to-spacing ratio (VDSR) of 2.98 ± 0.20 and a mean spacing of 2.93 ± 0.34 cm. The mean VGR and PVDR were 6.6 ± 1.9% and 4.36 ± 0.53, respectively. According to the Pearson correlation results, VDSR was not significantly correlated with either MVD (r = –0.38) or GTV size (r = –0.15). Due to the irregular geometry of recurrent GBM targets, vertex diameter was often constrained by regional GTV shape—particularly in strip-like or star-shaped volumes. Conceptually, if the GTV were modeled as a “LEGO” structure, which could be segmented into 2–4 cm pieces, the geometry of each piece determined its optimal vertex size. Consequently, a fixed-diameter vertex matrix was not suitable for all cranial cases. Because MVD determined VTV size, VGR was also largely constrained by GTV geometry. If the GTV could accommodate larger vertices (12–15 mm), the VTV size increased without reducing vertex spacing, making treatment objectives easier to achieve. A comparison between two typical cases illustrates this effect in **Figures 1A, B**. Although the GTV volumes for P1 and P5 were similar (43.1 cc and 48.6 cc, respectively), the smaller average segment size in P1 resulted in an MVD of 8.8 mm, compared to 12.2 mm in P5. Overall, no universal set of VTV parameters could be applied to all patients; VTV contours needed to be individualized based on GTV geometry.

For Group 2 patients, mean VGR and PVDR were 25.8 ± 10.0% and 5.01 ± 0.82, respectively demonstrating that the Group 2 planning approach could substantially increase the VGR compared to Group 1. An example case (P12) is presented in **Figure 1C**.

Across all patients, mean VTV coverage (V15Gy) was 95.33 ± 1.69%. The maximum dose to 0.1 cc of the PTV (D0.1cc) did not exceed 20 Gy in any plan.

### 3.2 SFRT course evaluation

GTV and PTV coverage with the 2nd course prescription in both SFRT and the original 2nd course plans are summarized in **Supplementary Table S3**. In 8 patients whose original prescriptions were ≤ 5 Gy/fraction, PTV coverage in the SFRT course matched the original plan within 0.41 ± 3.05%. This demonstrated that when no conflict existed between PTV prescription and valley dose constraints, SFRT could achieve valley dose objectives without compromising PTV coverage.

**Figure 2:**
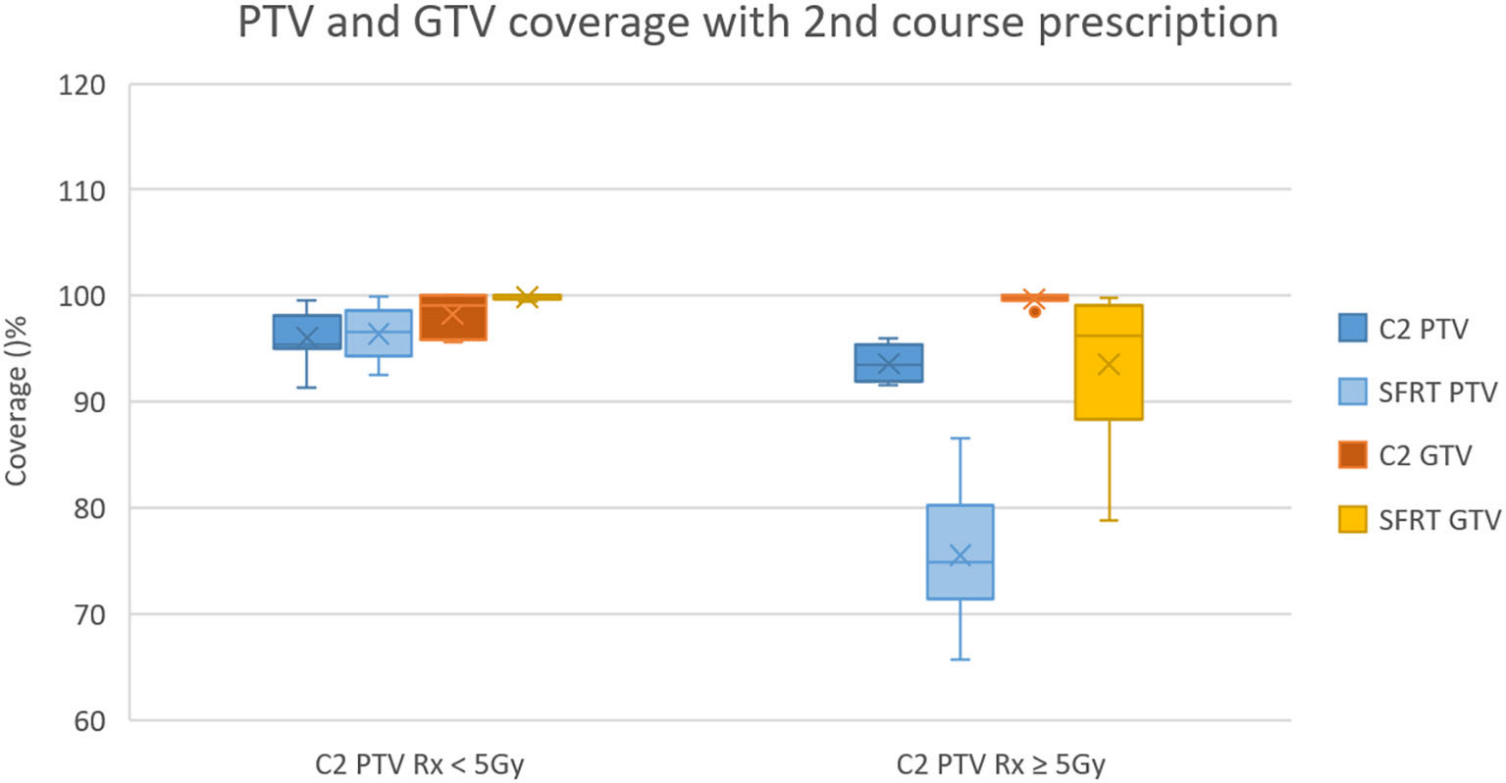
For the 8 patients whose original prescription was less than 5 Gy/fx (C2 PTV Rx < 5 Gy; left), PTV and GTV coverage in the SFRT course were 96.44 ± 2.57% and 99.88 ± 0.24%, respectively. For the 6 patients who received a downscaled prescription of 4 Gy/fx (C2 PTV Rx ≥ 5 Gy; right), PTV and GTV coverage in the SFRT course were 75.60 ± 6.79% and 93.58 ± 7.86%, respectively. C2 refers to the second course.

In the remaining 6 patients, whose SFRT plans used a downscaled 4 Gy PTV prescription, coverage in the SFRT course was 75.60 ± 6.79% for the PTV and 93.58 ± 7.86% for the GTV—both lower than in the original 2nd course plans, as expected. Coverage comparisons are shown in **Figure 2**.

### 3.3 SFRT sum plan evaluation

**Figure 3:**
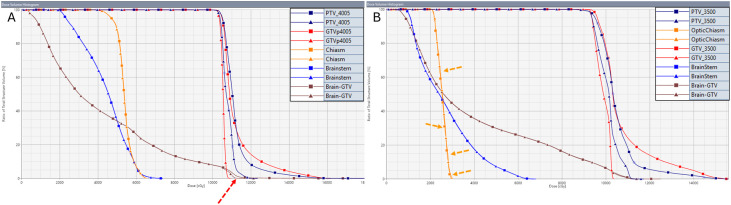
DVH plots of normal brain, brainstem, chiasm, GTV, and PTV for P1 **(A)** and P5 **(B)** in the SFRT (square markers) and original (triangle markers) sum plans. When the DVH γ pass rate exceeds 90%, discrepancies between the two DVHs are generally difficult to detect. For example, the chiasm DVH γ pass rate of P5 was 73.8%, showing small but continuous deviations (indicated by orange arrows). The DVH γ pass rate for the normal brain was 87.9% for P1 (discrepancy indicated by red arrows) and 98% for P5. This also illustrates that high-dose spillover to the normal brain was greater in P1 than in P5.

Accumulated EQD2 values for OARs and targets were compared between SFRT and original sum plans in **Supplementary Tables S4, S5**. The treatment plan records for the 1st course for P10 (in Group 1) were not available; therefore, this patient was excluded from all cumulative dose analyses. For the brainstem, the mean D0.03cc difference in the SFRT sum plan was 0.36± 1.00% compared to the original sum plan. Among eight patients with a dose increase, the mean increase was 0.59 ± 0.60% with a mean DVH γ pass rate of 98.1 ± 3%. When the DVH γ pass rate fell below 90%, visual inspection revealed the dose–volume discrepancy (e.g., P1 and P5 in **Figure 3**). One patient (P8) had a 2.8% brainstem dose increase and a DVH γ pass rate of 26.3%, attributed to GTV proximity to the brainstem.

For the optic chiasm, mean D0.03cc difference was –0.71± 3.53%. 6 patients showed slightly higher chiasm doses in the SFRT sum plan (mean increase 0.85 ± 0.60%, DVH γ pass rate 89.7 ± 4.6%). One patient (P8) had a 4.6% increase due to GTV encompassing half of the left optic nerve and chiasm. Overall, these results demonstrated that SFRT could maintain OAR sparing even when targets were adjacent to critical structures.

For Group 1, the D0.1cc dose to normal brain in the SFRT plan was 13.6 ± 11.8% higher than in the original plan, due to unavoidable high-dose spillover from the VTV. In contrast, for Group 2, the ≥ 5 mm margin between PTV and VTV allowed normal brain dose reduction, with a mean difference of –1.4 ± 2.8% compared to the original sum plan.

Across all patients, mean GTV DVH γ pass rate was 55.0 ± 12.4%, depending on vertex distribution. Mean dose escalation (D0.01cc) in the SFRT sum plan was 26.7 ± 9.2% for the PTV and 35.7 ± 8.9% for the GTV. Containing the entire VTV within the GTV (e.g., P5) effectively minimized normal brain spillover (**Figure 3**; **Supplementary Table S4**). Maintaining ≥ 1 cm distance between VTV and critical OARs allowed for improved sparing (e.g., P8, **Supplementary Table S4**).

### 3.4 Brain dose constraints and safety considerations

**Figure 4:**
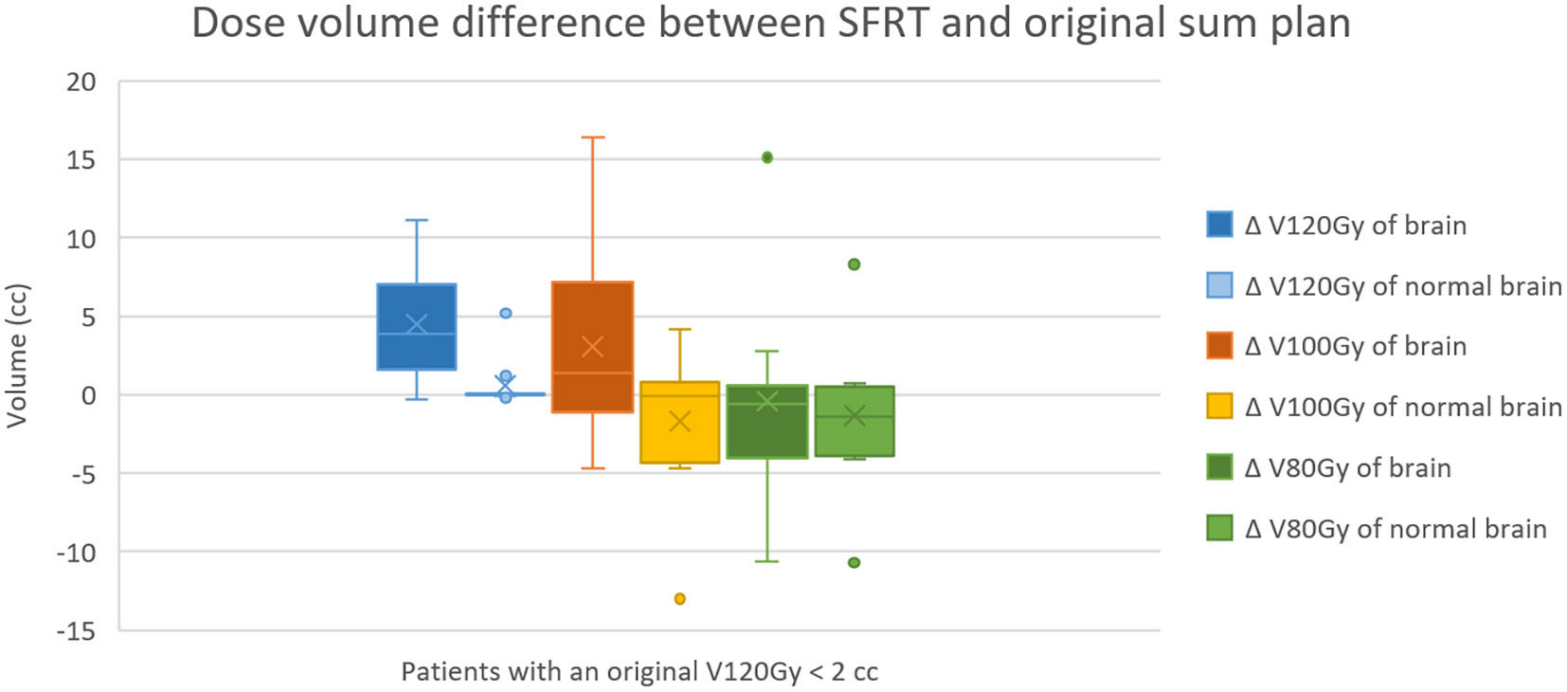
Statistical analysis of dose–volume differences between the SFRT and original sum plans for 12 patients whose original cumulative V120Gy in the brain was less than 2 cc. In the SFRT sum plan, V120Gy of the brain and normal brain increased by 4.51 ± 3.97 cc and 0.58 ± 1.58 cc, respectively. V100Gy of the brain and normal brain increased by 3.08 ± 6.31 cc and decreased by 1.69 ± 4.50 cc, respectively. V80Gy showed minimal changes, with differences of −0.41 ± 6.22 cc for the brain and −1.34 ± 4.54 cc for the normal brain.

For brain reirradiation, treatment is generally considered safe if cumulative EQD2 to brain, brainstem, and chiasm remains below 120 Gy, 100 Gy, and 75 Gy, respectively (26). V120Gy, V100Gy, and V80Gy values for brain and normal brain in both sum plans are shown in **Figure 4** and **Supplementary Table S6**.

In 11 patients with baseline V120Gy < 2 cc in the original sum plan, SFRT increased V120Gy by 4.51 ± 3.97 cc for brain and by 0.58 ± 1.58 cc for normal brain. Given the escalated peak dose in SFRT, this increase was expected and may be acceptable for aggressive, poor-prognosis diseases such as recurrent GBM. Physician judgment is essential to balance potential benefits against the risk of radiation induced necrosis. If the brain dose constraints are prioritized, reducing the SFRT peak dose to 10–12 Gy could serve as a compromise. For these 11 patients, V100Gy increased by 3.08 ± 6.31 cc for brain and decreased by 1.69 ± 4.50 cc for normal brain. Changes in V80Gy were minimal (–0.41 ± 6.22 cc for brain, –1.34 ± 4.54 cc for normal brain).

Two patients (P3 and P6) had very high baseline V120Gy values (76.3 cc and 88.4 cc) in the original sum plan. In these cases, SFRT would raise V120Gy above 100 cc, warranting caution. For such patients, prescription adjustments—such as reducing the peak dose or lowering total PTV dose—should be considered to mitigate risk.

## 4 Discussion

Our results demonstrated that a feasible SFRT plan could be achieved using a personalized VTV, suggesting a potential role for SFRT in expanding therapeutic options where conventional approaches might be limited. Nonetheless, several key considerations merit further discussion.

The GTV threshold of 15 cc was selected based on the practical feasibility of placing more than one vertex within the GTV. Specifically, this value falls between the smallest GTV in Group 1 (16.5 cc) and the largest GTV in Group 2 (10.6 cc), thereby serving as a natural dividing point within our patient cohort. In rare cases, a GTV smaller than 15 cc may still accommodate more than one vertex depending on its shape (e.g., a long, narrow geometry). However, the 15 cc threshold was chosen to reflect a consistent and broadly applicable criterion for this specific patient cohort.

In this dosimetric modeling study of SFRT for recurrent GBM, we showed that that dosimetrically acceptable plans, using SFRT as an upfront boost, can be achieved for recurrent GBM. We selected peak and valley SFRT doses that might optimize tumor control and normal tissue sparing. A peak dose of 15 Gy was selected as it represents a median ablative dose capable of enhancing tumoricidal effects, inducing vascular damage, and potentially triggering immunogenic responses. A valley dose of < 5 Gy was applied because it is considered non-ablative and unlikely to damage the vascular system, thereby maintaining perfusion for tumor-targeting immune cells and cytokines. In clinical practice, both peak and valley doses may be modified based on tumor control outcomes and cumulative OAR dose constraints.

For irregularly shaped cranial tumors, no universal set of vertex parameters can be applied to all cases. Script-generated vertices in this study still required manual adjustment for each patient. The modification process was time-consuming, requiring 5–20 minutes after each iteration of plan optimization. The total planning time largely depended on both the number of vertices and the planner’s level of proficiency. Future work will focus on revising the script to generate VTV contours more adaptively, reducing the need for manual intervention.

Vertex diameters between 8–15 mm were used in this study, reflecting both machine delivery constraints and GTV shape limitations. When MVD was < 8 mm, VDSR was increased to meet valley dose objectives, which often resulted in VGR values below 3%. In this cohort, VGR was limited by MVD, with a maximum observed value of 9.5%. Although the relationship between VGR and tumor control is not yet clearly established, increasing VGR without causing unacceptable OAR toxicity remains a key goal in SFRT planning. This limitation may vary across machines and delivery systems—for example, Gamma Knife or linac-plan with stereotactic cones may allow for smaller vertices and denser distribution to achieve similar dose objectives; however, delivery efficiency could be compromised if a large number of vertices are required.

A key distinction between extracranial and cranial SFRT targets is that, beyond their typical size differences, recurrent GBM lesions tend to exhibit much more irregular geometry. Conventional extracranial SFRT targets are often more spherical or bulky, which makes fixed-diameter vertex matrices more feasible. In contrast, the irregular, strip-like, or star-shaped cranial targets require greater flexibility. In this context, the “LEGO” approach of segmenting the GTV into smaller geometric pieces can help guide vertex design and placement more effectively. Such an approach allows vertex size and spacing to adapt to local geometry, which may be advantageous in achieving consistent valley-peak modulation while respecting anatomic constraints. Ultimately, GTV geometry determines the maximum feasible vertex diameter and achievable VGR.

Cumulative dose to OARs outside the GTV was not increased by more than 5% with SFRT in this study. Normal brain dose could be effectively minimized by ensuring that the VTV was entirely contained within the GTV. However, the elevated biologically effective dose (BED) associated with the SFRT peak dose introduces the risk of higher cumulative brain dose, necessitating a balance between maximizing tumor control and minimizing the risk of radiation necrosis. To mitigate this risk without taking on excessive toxicity, SFRT could be applied with a lower total course prescription or by using smaller VTV, albeit at the expense of reduced VGR and potentially lower tumor control rates. Based on the treatment guidelines from RTOG 0825 and RTOG 1205, a typical prescription for primary and recurrent treatments is 60 Gy in 30 fractions and 35 Gy in 10 fractions, respectively. This leaves approximately 10 Gy in a single fraction available before reaching a cumulative EQD2_8_ of 120 Gy. For the SFRT plan, a peak dose of 8–10 Gy and a valley dose of around 3.5 Gy can be selected, which helps minimize the risk of radiation necrosis. In this way, the radiobiological advantages of SFRT, including vascular damage within the peak-dose regions and an enhanced anti-tumor immune response, can be maintained while better preserving surrounding normal tissue.

Future research will include preclinical studies using rodent GBM models to investigate the relationship between tumor control and SFRT prescription parameters. In addition, given the growing evidence supporting the synergistic potential of SFRT and immunotherapy, exploring this combination represents another promising direction for radiobiological investigation (17, 27). Furthermore, development is underway on an SFRT plan evaluation script capable of analyzing VTV contours before planning. This tool is also designed to automatically generate plan-check reports with key SFRT parameters, including VTV dimensions, PVDR, VGR, and equivalent uniform dose (EUD) for the GTV and PTV.

## 5 Conclusion

This planning study demonstrated that dosimetrically acceptable SFRT plans can be achieved using vertex diameters of 0.8–1.5 cm and vertex spacing of 2–4 cm. Peak-to-valley dose ratios exceeding 3 were consistently obtained in this study. When the VTV was entirely contoured within the GTV and maintained a 2 mm margin, SFRT provided comparable or improved sparing of nearby OARs and normal brain tissue relative to conventional radiotherapy.

Future work will focus on automating SFRT planning procedures and investigating the relationship between VTV and tumor control rate. Moreover, the combination of SFRT with immunotherapy holds promise for enhancing therapeutic outcomes, warranting further preclinical and clinical evaluation.

## Data Availability

The original contributions presented in the study are included in the article/**Supplementary Material**. Further inquiries can be directed to the corresponding authors.
