# Supplementary material for: Spatially fractionated radiation therapy for treating recurrent glioblastoma: a dosimetric feasibility study
Source: Front Oncol. 2026 Jan 5;15:1691132. doi: 10.3389/fonc.2025.1691132 (PMC12813869; doi:10.3389/fonc.2025.1691132)
Supplement: Supplementary file 1 [file DataSheet1.pdf]

Table S1. Original prescription of each course for all patients

| | **Prescription** | | | **Recurrence patterns** |
|---|---|---|---|---|
| | 1st Course Rx | | 2nd Course Rx | |
| **P1** | 60 Gy/ 30 fx | | 40.05 Gy/15 fx | In-field |
| **P2** | 59.4 Gy/ 33 fx | | 40.05 Gy/15 fx | In-field |
| **P3** | 60 Gy/ 30 fx | | 50 Gy/ 20 fx | In-field |
| **P4** | 60 Gy/ 30 fx | | 30 Gy/ 5 fx | In-field |
| **P5** | 60 Gy/ 30 fx | | 35 Gy/ 10 fx | In-field |
| **P6** | 60 Gy/ 30 fx | | 59.4 Gy/ 33 fx | In-field |
| **P7** | 60 Gy/ 30 fx | 25 Gy/ 5 fx | 25 Gy/ 5 fx | Out-of-Field |
| **P8** | 60 Gy/ 30 fx | | 40.05 Gy/15 fx | Out-of-Field |
| **P9** | 60 Gy/ 30 fx | | 25 Gy/ 5 fx | In-field |
| **P10** | 59.4 Gy/ 33 fx | 20 Gy/ 1 fx | 35 Gy/ 5 fx | Marginal |
| **P11** | 60 Gy/ 30 fx | | 40.05 Gy/15 fx | Marginal |
| **P12** | 60 Gy/ 30 fx | | 30 Gy/ 5 fx | Marginal |
| **P13** | 60 Gy/ 30 fx | | 35 Gy/ 10 fx | Marginal |
| **P14** | 60 Gy/ 30 fx | | 30 Gy/ 5 fx | In-field |

*6 patients using a downscaled 4Gy/fx prescription for PTV in SFRT plan were highlighted.

In-field: D95% of recurrence volume ≥ 95% Rx (Rx = 46, 50 or 54Gy, according to the prescription of initial target volume in first course)

Marginal: 20% Rx <D95% of recurrence volume <95% Rx

Out-of-Field: D95% of recurrence volume < 20% Rx

Table S1 summarizes the original prescription details for both the first and second courses of treatment for each patient. All primary treatments followed the RTOG 0825 protocol, using either 60 Gy in 30 fractions or 59.4 Gy in 33 fractions. For recurrent disease, various techniques were employed, as there is currently no established consensus on the optimal re-irradiation approach.

Recurrence patterns were categorized as follows:

1. In-field: The recurrent tumor (GTV in the second course) is located within the high-dose region of the initial treatment, defined as having D95% of the recurrence volume ≥ 95% of the original prescription dose (Rx = 46, 50 or 54Gy, according to the prescription of initial target volume in first course).

2. Marginal: The recurrence occurs at the edge of the high-dose radiation field, where D95% of the recurrence volume falls between 20% and 95% of the original prescription dose.
3. Out-of-field: The recurrence arises entirely outside the original radiation field, with D95% of the recurrence volume < 20% of the original prescription dose.

Patients P7 and P10 each underwent three courses of treatment. However, the planning target volumes (PTVs) in their second and third courses did not overlap. Therefore, for the purpose of analysis, the first and second courses were combined and considered as the original (first) course, while the third course was treated as the second course.

For patient P10, detailed treatment records—including DICOM files—for the primary course (59.4 Gy/ 33 fx) were unavailable. Relevant information was instead extracted from a special consultation note.

Table S2. Optimized vertices and SFRT plan parameters

| | MVD (mm) | MVS (mm) | VDSR | VTV (cc) | GTV (cc) | VTV to GTV ratio | V15Gy of VTV | Peak dose (cGy) | Valley dose (cGy) | PVDR |
|---|---|---|---|---|---|---|---|---|---|---|
| **P1** | 8.9 | 26.8 | 3.03 | 2.65 | 43.1 | 6.1% | 96.7% | 1499.6 | 298.9 | 5.02 |
| **P2** | 8.8 | 27.6 | 3.13 | 1.98 | 25.2 | 7.9% | 95.2% | 1648.2 | 369 | 4.47 |
| **P3** | 10.0 | 30.5 | 3.05 | 3.94 | 43.7 | 9.0% | 94.7% | 1651.4 | 344.5 | 4.79 |
| **P4** | 10.2 | 27.2 | 2.66 | 2.55 | 47.9 | 5.3% | 96.7% | 1526.9 | 395.5 | 3.86 |
| **P5** | 12.2 | 35.6 | 2.92 | 4.64 | 48.6 | 9.5% | 97.1% | 1795.7 | 365.7 | 4.91 |
| **P6** | 9.0 | 24.7 | 2.75 | 1.87 | 22.6 | 8.3% | 95.9% | 1640.2 | 337.3 | 4.86 |
| **P7** | 8.3 | 27.0 | 3.24 | 0.89 | 16.5 | 5.4% | 95.7% | 1524.7 | 429.9 | 3.55 |
| **P8** | 9.7 | 31.6 | 3.25 | 3.26 | 60.2 | 5.4% | 91.3% | 1482.7 | 355.5 | 4.17 |
| **P9** | 11.4 | 33.0 | 2.91 | 6.46 | 117.8 | 5.5% | 94.1% | 1528.1 | 361.9 | 4.22 |
| **P10** | 10.0 | 28.5 | 2.85 | 1.48 | 41.6 | 3.6% | 95.9% | 1353.6 | 359 | 3.77 |
| **P11** | — | — | — | 1.31 | 3.7 | 35.4% | 96.8% | 1480.9 | 254.9 | 5.81 |
| **P12** | — | — | — | 1.71 | 8.2 | 20.9% | 97.3% | 1734.8 | 407.5 | 4.26 |
| **P13** | — | — | — | 2.25 | 6.2 | 36.3% | 97% | 1821.2 | 419.8 | 4.34 |
| **P14** | — | — | — | 1.15 | 10.6 | 10.8% | 93.6% | 1639.9 | 291.7 | 5.62 |

*MVD: Mean Vertices Diameter; MVS: Mean Vertices center-to-center Spacing; VDSR: Vertices Diameter to Spacing Ratio; PVDR: Peak to Valley Dose Ratio

Table S3. PTV and GTV coverage with 2nd course prescription

| | **Coverage of 100% C2 Rx** | | | | | **Coverage of 95% C2 Rx** | |
|---|---|---|---|---|---|---|---|
| | PTV | | | GTV | | PTV | GTV |
| | C2 (%) | SFRT (%) | Diff (%) | C2 (%) | SFRT (%) | SFRT (%) | SFRT (%) |
| **P1** | 95 | 99.9 | 4.9 | 95.6 | 100 | | |
| **P2** | 95.6 | 95.6 | 0 | 100 | 99.4 | | |
| **P3** | 91.3 | 94.4 | 3.1 | 98.6 | 100 | | |
| **P4** | 93.5 | 74.8 | -18.7 | 99.9 | 91.6 | 89.3 | 99.5 |
| **P5** | 98.2 | 94.3 | -3.9 | 99.6 | 99.6 | | |
| **P6** | 95.3 | 92.6 | -2.7 | 96.7 | 100 | | |
| **P7** | 92 | 86.6 | -5.4 | 99.9 | 94.5 | 94.8 | 100 |
| **P8** | 97.9 | 97.6 | -0.3 | 100 | 100 | | |
| **P9** | 93.4 | 65.74 | -27.66 | 98.5 | 78.8 | 96.2 | 99.3 |
| **P10** | 96 | 74.95 | -21.05 | 100 | 98 | 88.6 | 100 |
| **P11** | 99.6 | 98.7 | -0.9 | 100 | 100 | | |
| **P12** | 91.6 | 73.4 | -18.2 | 99.9 | 98.8 | 91.85 | 100 |
| **P13** | 95.3 | 98.4 | 3.1 | 100 | 100 | | |
| **P14** | 95.2 | 78.1 | -17.1 | 100 | 99.8 | 91.2 | 100 |

*Rx: prescription; C2: original 2nd course

6 patients used a downscaled 4Gy/fx prescription for PTV in SFRT plan were highlighted.

Table S4. Accumulative EQD2 and DVH gamma analysis of OARs

| | **Normal brain** | | | | **Brainstem** | | | | **Chiasm** | | | |
|---|---|---|---|---|---|---|---|---|---|---|---|---|
| | D0.1cc (Gy) | | | | D0.03 (Gy) | | | | D0.03 (Gy) | | | |
| | Original | SFRT | Diff % | DVH γ | Original | SFRT | Diff % | DVH γ | Original | SFRT | Diff % | DVH γ |
| **P1** | 114.2 | 155.8 | 36.4% | 87.9% | 70.6 | 70.8 | 0.2% | 96.0% | 61.3 | 61.4 | 0.2% | 90.9% |
| **P2** | 110.5 | 134.6 | 21.8% | 86.0% | 29.0 | 29.0 | 0.1% | 100.0% | 13.4 | 12.4 | -7.4% | 88.4% |
| **P3** | 127.1 | 156.1 | 22.8% | 83.0% | 69.6 | 70.1 | 0.7% | 98.3% | 61.6 | 62.1 | 0.8% | 86.1% |
| **P4** | 121.1 | 131.6 | 8.7% | 63.7% | 56.1 | 55.6 | -1.0% | 88.0% | 11.9 | 11.6 | -1.9% | 61.7% |
| **P5** | 111.9 | 117.4 | 4.9% | 98.0% | 64.9 | 65.6 | 1.1% | 100.0% | 27.7 | 25.4 | -8.4% | 73.8% |
| **P6** | 124.2 | 145.5 | 17.1% | 97.7% | 62.7 | 63.0 | 0.4% | 100.0% | 60.2 | 61.2 | 1.5% | 83.9% |
| **P7** | 130.7 | 130.0 | -0.5% | 90.2% | 71.5 | 71.1 | -0.5% | 92.8% | 59.3 | 59.0 | -0.5% | 88.6% |
| **P8** | 112.6 | 120.5 | 7.0% | 85.3% | 57.4 | 59.0 | 2.8% | 26.3% | 31.9 | 33.4 | 4.6% | 72.3% |
| **P9** | 112.5 | 116.8 | 3.9% | 60.3% | 87.2 | 86.6 | -0.7% | 100.0% | 68.3 | 68.5 | 0.3% | 95.7% |
| **P10** | 187.9 | 187.8 | 0.0% | 53.7% | 8.0 | 7.1 | -11.4% | 37.8% | 16.6 | 14.9 | -10.1% | 44.3% |
| **P11** | 107.6 | 106.1 | -1.4% | 76.4% | 48.8 | 48.7 | -0.1% | 100.0% | 34.3 | 34.5 | 0.6% | 87.6% |
| **P12** | 118.6 | 114.5 | -3.4% | 69.2% | 64.7 | 64.7 | 0.0% | 100.0% | 37.2 | 37.8 | 1.6% | 93.7% |
| **P13** | 103.4 | 106.0 | 2.6% | 83.9% | 33.0 | 33.5 | 1.6% | 92.2% | 4.3 | 4.2 | -0.2% | 77.3% |
| **P14** | 120.3 | 116.4 | -3.3% | 67.2% | 63.1 | 63.1 | 0.0% | 100.0% | 40.3 | 40.1 | -0.4% | 91.3% |

*Original: Original sum plan; SFRT: SFRT sum plan

DVH gamma analysis used equation 5 in reference 16. In this study, we ignore the DVH above D0.1cc in SFRT sum plan for GTV, PTV, brain and normal brain. As the DICOM files for the 59.4 Gy in 33 fractions treatment course of patient P10 are not available, comprehensive dose accumulation analysis could not be performed. Therefore, the dose analysis for P10 only demonstrates that the SFRT technique did not increase the dose to organs at risk (OARs). However, cumulative radiation toxicity could not be assessed for this patient.

Table S5. Accumulative EQD2 of PTV and GTV

| | PTV | | | GTV | | |
|---|---|---|---|---|---|---|
| | D0.1cc | | | D0.1cc | | |
| | Original | SFRT | Diff | Original | SFRT | Diff |
| **P1** | 120.4 | 159.1 | 32.1% | 108.2 | 160.0 | 61.9% |
| **P2** | 111.4 | 150.2 | 34.9% | 105.3 | 150.2 | 42.7% |
| **P3** | 126.3 | 165.5 | 31.1% | 123.1 | 165.4 | 34.3% |
| **P4** | 121.6 | 155.2 | 27.6% | 121.1 | 155.3 | 28.2% |
| **P5** | 111.9 | 153.5 | 37.2% | 103.4 | 153.5 | 48.5% |
| **P6** | 124.2 | 145.5 | 17.1% | 124.3 | 170.3 | 36.9% |
| **P7** | 85.9 | 102.6 | 19.3% | 85.2 | 130.0 | 52.6% |
| **P8** | 112.6 | 146.4 | 30.1% | 107.4 | 146.5 | 36.4% |
| **P9** | 115.8 | 139.0 | 20.0% | 98.8 | 139.9 | 41.7% |
| **P10** | 93.0 | 108.8 | 17.0% | 70.8 | 108.8 | 53.8% |
| **P11** | 98.5 | 102.7 | 4.3% | 58.4 | 76.5 | 31.0% |
| **P12** | 110.4 | 136.5 | 23.6% | 105.6 | 136.5 | 29.2% |
| **P13** | 103.4 | 130.9 | 26.6% | 91.3 | 130.9 | 43.4% |
| **P14** | 123.6 | 153.6 | 24.3% | 123.6 | 153.6 | 24.3% |

*Original: Original sum plan; SFRT: SFRT sum plan; Diff: percentage difference

As the DICOM files for the 59.4 Gy in 33 fractions treatment course of patient P10 are unavailable, the dose analysis for this patient can only demonstrate that the SFRT technique increased the dose to the PTV and GTV as expected.

Table S6. V120Gy, V100Gy and V80Gy of brain and normal brain in original and SFRT sum plans

| | V120Gy of Brain (cc) | | | | V100Gy of Brain (cc) | | | | V80Gy of Brain (cc) | | | |
|---|---|---|---|---|---|---|---|---|---|---|---|---|
| | Brain | | Normal brain | | Brain | | Normal brain | | Brain | | Normal brain | |
| | Original | SFRT | Original | SFRT | Original | SFRT | Original | SFRT | Original | SFRT | Original | SFRT |
| **P1** | 0 | 11.1 | 0 | 5.2 | 160.1 | 159 | 121.5 | 120.4 | 240.8 | 241 | 202.2 | 202.4 |
| **P2** | 0 | 5.5 | 0 | 1.2 | 146.6 | 147.8 | 122.1 | 123.1 | 226.1 | 226.7 | 201.4 | 201.9 |
| **P3** | 76.3 | 150.7 | 72.1 | 114.4 | 269.5 | 273.4 | 226.6 | 230.5 | 397.1 | 401.3 | 354.2 | 358.4 |
| **P4** | 0.4 | 10.8 | 0.2 | 0.4 | 77.5 | 72.8 | 31.7 | 27 | 117.1 | 113.1 | 70.3 | 66.4 |
| **P5** | 0 | 6.8 | 0 | 0.02 | 118.5 | 134.9 | 97.5 | 97.4 | 297.3 | 295.4 | 249.3 | 247.3 |
| **P6** | 88.4 | 101.8 | 67.1 | 79.8 | 213.5 | 213.1 | 191.5 | 191 | 336 | 334.5 | 314 | 312.4 |
| **P7** | 2 | 1.7 | 1.9 | 1.7 | 5.5 | 5.5 | 5.5 | 5.4 | 11.6 | 11 | 11.3 | 10.8 |
| **P8** | 0 | 1.6 | 0 | 0.1 | 48.7 | 55.9 | 47.1 | 51.3 | 148.3 | 163.4 | 143.5 | 151.8 |
| **P9** | 0 | 7 | 0 | 0.1 | 28.8 | 39 | 28.8 | 15.8 | 281.3 | 270.7 | 168.8 | 158.1 |
| **P10** | — | — | — | — | — | — | — | — | — | — | — | — |
| **P11** | 0 | 0 | 0 | 0 | 4 | 5.4 | 4 | 4.8 | 15.1 | 17.9 | 14.9 | 15.6 |
| **P12** | 0 | 1.9 | 0 | 0 | 8.8 | 11.2 | 7.2 | 5.3 | 30.2 | 28.5 | 22.7 | 20.8 |
| **P13** | 0 | 1.7 | 0 | 0 | 0.6 | 5.8 | 0.6 | 1.2 | 34.5 | 34.2 | 28.5 | 28.1 |
| **P14** | 1.2 | 5.1 | 0.1 | 0 | 32.4 | 28.1 | 22.5 | 18.2 | 57.8 | 53.7 | 47.9 | 43.8 |

*Original: Original sum plan; SFRT: SFRT sum plan

As the DICOM files for the 59.4 Gy in 33 fractions treatment course of patient P10 are unavailable, brain toxicity analysis could not be performed for this patient.

The higher normal brain doses observed in patients P3 and P6 are primarily attributable to the second-course prescription (EQD2 ≈ 60 Gy) and the 5 mm PTV margin applied for GBM re-irradiation. The regions with EQD2 values exceeding 120 Gy are largely confined to the PTV margin, which consequently contributes to the increased dose observed in the adjacent normal brain tissue.
